# Type 2 diabetes and physical activity: barriers and enablers to diabetes control in Eastern India

**DOI:** 10.1017/S146342361800097X

**Published:** 2019-04-29

**Authors:** S. Pati, E. Lobo, S. Pati, S. Desaraju, P. Mahapatra

**Affiliations:** 1 ICMR – Regional Medical Research Centre, Department of Health Research, Government of India, Bhubaneswar, Odisha, India; 2 Indian Institute of Public Health Bhubaneswar, Public Health Foundation of India, Bhubaneswar, Odisha, India; 3 Directorate of Public Health, Department of Health and Family Welfare, Government of Odisha, Bhubaneswar, Odisha, India; 4 Department of Psychiatry, Kalinga Institute of Medical Sciences, KIIT University, Bhubaneswar, Odisha, India

**Keywords:** diabetes control, India, internal and external barriers, patient activation, personal enablers

## Abstract

**Introduction:**

Type 2 diabetes (T2D) has tremendous morbidity burden owing to disease management and complication prevention. Behavior modification identified as a key to management includes healthy diet and regular physical activity (PA). This study aims to identify patterns and preferences of PA of T2D patients and explore perceived enablers and barriers for diabetes control in Bhubaneswar.

**Methods:**

Cross-sectional, facility-based study conducted in the private sector from June to August 2014 recruited 321 T2D patients using semi-structured questionnaires. Descriptive statistics and associations of PA were computed.

**Results:**

Almost two-thirds of patients (59%) were reported performing PA frequently. Majority patients cited walking as the most preferred mode of PA (79%) with 41% performing PA daily. Actual versus perceived weight was a complete mismatch with most patients misjudging their weight. Reasons for enabling PA included ‘controlling diabetes’ and ‘doctor’s advice’ as key factors, while ‘lack of time’ and ‘unwillingness’ were main barriers among inactive patients.

**Conclusion:**

Counseling on PA by physicians during routine visits, along with tailored or patient-specific interventions should be considered. Focus on social support for positive behavioral changes and motivation play a central role in diabetes control.

## Introduction

A total of 442 million people worldwide live with diabetes, and type 2 diabetes (T2D) comprises 90% of the burden (Huffman and Vaccaro, 2012; World Health Organization, 2016). India is referred to as the ‘Diabetes Capital of the World’ since the International Diabetes Federation has stated that the number of diabetics is expected to rise from 40.9 million to 69.9 million by 2025 (Madaan *et al.*, 2014). Besides genetic predisposition to diabetes, studies have also shown strong linkage to four key behavioral risk factors, viz., tobacco consumption, physical inactivity, unhealthy diet, and increased use of alcohol (World Health Organization, 2016). Although the pandemic of physical inactivity causes 7% of the burden of disease from diabetes and is attributed to 9% of premature mortality (Lee *et al.*, 2012), the rising prevalence of diabetes largely driven by physical inactivity among other factors has become a major concern for healthcare in India (World Health Organization, 2014).

Physical activity (PA) not only contributes to prevention or delay in development of other long-term diabetes complications, such as neuropathy, retinopathy, and nephropathy, but also may slow the progression of existing complications (Thomas *et al*., 2004). Additionally, it also includes positive impact on insulin action, glycemic control, and metabolic abnormalities associated with T2D (Paffenbarger *et al*., 1993; Pate *et al*., 1995; Hayes and Kriska, 2008). Thus based on clear evidences that PA is essential for the management of T2D (Pan *et al*., 1997; Tuomilehto *et al.*, 2001; Knowler *et al*., 2002), physicians treating diabetes patients generally advise uptake or increase in PA levels, consumption of a healthy diet, and, if needed, tablets and/or insulin to control blood glucose levels (Lawton *et al.*, 2006). Thus, for self-management beyond clinical treatment, the onus lies on the patient. However, studies have reported that individuals with diabetes engage in less PA than non-diabetics, live more sedentary lifestyles, and have poor metabolic control (Ford and Herman, 1995; Olivarius *et al*., 2001; Nelson *et al*., 2002). This perhaps can be attributed to various personal, environmental, psychosocial factors that may interfere with following exercise recommendations, thus making diabetes management difficult (Huffman and Vaccaro, 2012). The principles of Alma-Ata declaring ‘Health for All’ though not directly, but indeed signify major importance with context to control and management of diabetes as well (World Health Organization, 1978). Thus, our study aims to assess the pattern and proportion of PA among T2D patients, and the associated enablers and barriers for management of diabetes in Eastern India. The identified enablers and barriers then in turn can be examined at patient, community, and perhaps even at policy-level for adopting changes for management of the diabetes pandemic.

## Methods

A cross-sectional, facility-based study with 341 T2D patients recruited from specialist clinics at a private hospital in Bhubaneswar, Odisha from June to August 2014 was conducted. The facility selection was done by purposive sampling due to high case load and study feasibility.

Using OpenEpi version 3, the minimum required sample size was calculated as 321 by means of the formula


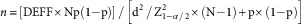


where *n*=the required sample size; population size (*N*)=100,000; *p*=hypothesized frequency – 25%; *d*=confidence limits – 5%; DEFF=design effect – 1.

From the above formula the sample size selected was 289. Considering 10% non-response rate, the sample size calculated was 289/(1 − 0.1). Hence the sample size finalized was 321.

Face-to-face interviews were conducted using a semi-structured questionnaire whose domains included socio-demographic details as well as information related to diabetes, and pattern of PA. Each participant (patient) was included only once in the study during the study period, after obtaining verbal consent for participation.

Data entry and analysis was done with Statistical Package for the Social Sciences (SPSS) version 20, to obtain descriptive statistics. Categorical data were presented as frequency (%). *χ* ^2^-test was used to identify the association between socio-demographic factors and pattern of PA.

The study was reviewed and approved by the Institutional Ethics Committee of Indian Institute of Public Health Bhubaneswar (IIPH-B). Patient anonymity was maintained through assigning of unique codes and data were kept confidential.

## Results

### Patient demographics and characteristics

As depicted in Table 1, among the 321 T2D patients interviewed, above 60% patients were between 35 and 60 years (*n*=196) with mean age of 51 years with 12.8 SD. Similarly, 64% (*n*=204/321) were males and proportion of graduates was the highest (44%), while 54% contributed to family income. Table 1 also shows 61% patients (*n*=195) consumed non-vegetarian food as their primary diet, while 8% reported smoking and 28% consuming smokeless tobacco; 55% patients admitted to family history of diabetes, while 69% patients reported spouse suffering from diabetes. Almost 70% of diabetes patients (69%, *n*=220) reported co-morbidities: hypertension, arthritis, gastritis, asthma, and cardiovascular diseases.

**Table 1:** Patient profile (*N*=321)

	*n* (%)
Age (in years)	
20–34	44 (14)
35–60	81 (25)
>60	196 (61)
Mean (SD)	51 (12.8 years)
Gender	
Male	204 (64)
Female	117 (36)
Education	
Uneducated	9 (3)
Secondary and below	93 (29)
Higher secondary	18 (6)
Graduate	140 (44)
Postgraduate and above	61(19)
Income contribution	
Housewife	96 (30)
Contributing	172 (54)
Not contributing	46 (14)
Not available	7 (2)
Food habits	
Vegetarian	126 (39)
Non-vegetarian	195 (61)
Smoker	
Yes	27 (8)
No	294 (92)
Smokeless tobacco user	
Yes	91 (28)
No	230 (72)
Family history of diabetes	
Yes	176 (55)
No	145 (45)
Spouse history of diabetes	
Yes	55 (17)
No	266 (83)
Co-morbid conditions	
Yes	220 (69)
No	101 (32)
Type of co-morbid conditions^1^	
Hypertension	162
Arthritis	48
Gastritis	46
Asthma	19
Cardiovascular disease	17

1 Some patients reported more than one co-morbid condition

### Diet and PA

From the entire cohort of T2D patients, 45% (*n*=144/321) reported strict control of their diet, 44% patients reported controlling their diet moderately, while 11% did not practice any form of diet control.

Further almost 60% patients reported (*n*=190/321; 59%) performing PA frequently. These patients were categorized as active and the remaining 41% were categorized as inactive since they reported of not performing any kind of PA. Gender differences showed that 62% males (*n*=127/204) performed PA compared to 54% females (*n*=63/117) (data not shown).

**Figure 1:**
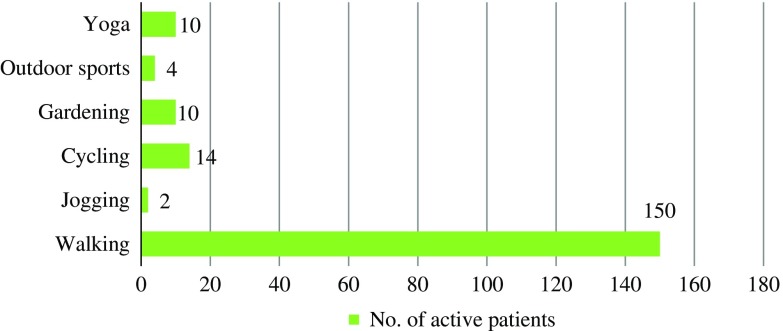
Frequently undertaken physical activities as reported by 190 type 2 diabetes patients. The figure depicts various types of physical activities undertaken by the ‘active’ patients in the cohort, with walking as the most preferred activity

Regarding the forms of PA undertaken, as seen in Figure 1, almost 80%, that is, 150 (79%; *n*=150/190) patients undertook walking – either morning or any time of the day, 7% reported cycling, 5% each reported gardening and yoga, four patients undertook outdoor sports, and two patients preferred jogging.

Of the 190 ‘active’ patients, three-fourths stated that they started exercising after being diagnosed with diabetes (*n*=142/190; 75%), while the remaining 25% reported that they were in the habit of exercising before their diabetes diagnosis (*n*=48) (data not shown).

Further as seen in Table 2, more than 50% patients reported that they were exercising 2–3 times per week (51%; *n*=97/190), while 41% patients were exercising daily, and <5% patients were exercising once a week or less. Duration of PA was variable between patients, with almost two-thirds of patients (59%, *n*=112) performing PA for <30 min per session compared to almost similar number of patients that performed PA for more than 30 and 45 min, respectively.

**Table 2:** Frequency and duration of performing physical activities for diabetes management and control as reported by T2D patients (*N*=190)

	*n* (%)
Frequency of physical activity	
Once a week	7 (4)
2–3 times per week	97 (51)
4–5 times per week	8 (4)
Daily	78 (41)
Duration of physical activity	
<30 min	112 (59)
30–45 min	37 (19)
>45 min	41 (22)

### Perception regarding own weight

**Figure 2:**
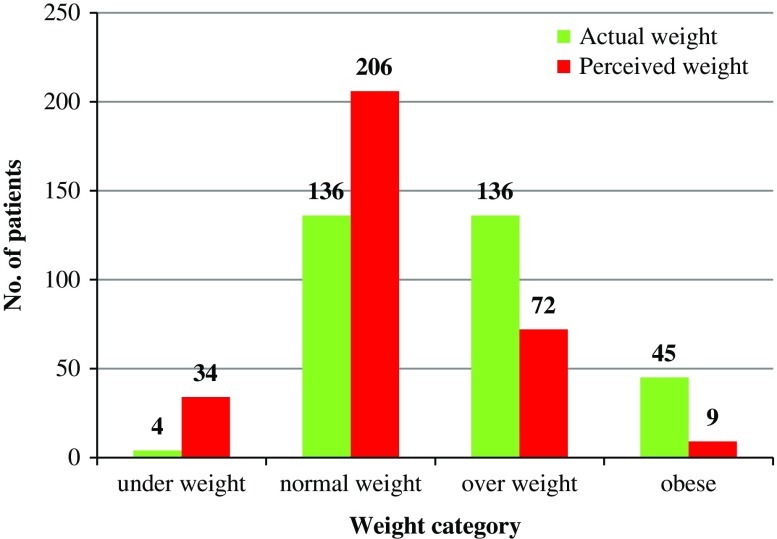
Distribution of perceived and actual weight of the 321 type 2 diabetes patients. The figure represents the mismatch of ‘perceived’ versus ‘actual’ weight of the entire cohort. The assumption of weight category by each patient was done prior to measuring individual weight

After patients were weighed, each patient was questioned to assume their weight category. Of the total 321 patients, a complete mismatch of individual perceived and actual weights was seen. As seen in Figure 2, though only four patients were underweight, 34 patients perceived themselves as being underweight. Similar observations were seen for normal weight patients (136 versus 206). While for the overweight and obese categories, fewer patients underestimated their actual weight categories (72 versus 136 and 9 versus 45).

### Barriers and enablers to PA

Barriers and enablers, that is, reasons for not performing or performing PA were captured during the study. These reasons were divided into personal or *internal* reasons that could be controlled or dependent on the patients (Korkiakangas, Alahuhta and Laitinen, 2009), while *external* reasons were related to infrastructure, weather, and so on, or independent (Serour *et al.*, 2007). Our study documented both personal and external reasons contributed to enabling patients to perform PAs (Table 3).

**Table 3:** Reasons for performing physical activity, viz., barriers and enablers

Barriers^1^	*N*=131
*Personal*	
Age	4
Work at home	24
Unwillingness	67
‘*Feel fit*’	2
Laziness	30
Shy	6
Tired/discomfort	31
Illness including joint pain and pregnancy	34
Weakness	21
Fear of injury	1
*External*	
Climate	2
Lack of time/workload/other engagements	82
Lack of road safety and place for exercise	5
No company	4
No knowledge of type of exercise	1

Enablers^1^	*N*=190
*Personal*	
Diabetes control	90
Blood pressure control	14
Reduce joint pain	10
Weight management	25
‘*Feel good*’	65
Stay active/fitness	59
Increase appetite	23
‘*Get good sleep*’	5
*External*	
Doctor’s advice	55
Family insisted	10
Friend’s suggestion	4
Company for exercise	14
Dog walking	1

1 Some patients may have given more than one reason as an enabler or barrier

The key personal reasons for barriers of not performing PA included unwillingness by patients, laziness, illness, and tiredness/discomfort. Among the four patients that cited their age as a barrier for PA – all were above 70 years. Only two patients ‘*felt fit*’, but did not perform any PA. The most common external barriers included lack of time or workload and other engagements in majority of the patients. Five patients cited lack of safe road and place to exercise as well, as seen in Table 3.

Personal enablers included control of diabetes, ‘*to feel good*’ and stay active, while external enablers included doctors’ advice and company for exercise featured as prominent enablers. Patients also stated other important reasons for exercising such as controlling other illnesses, including hypertension and joint pain, along with weight management.

## Discussion

Our study describes the pattern of PA by T2D patients, and the enablers and barriers that influence their practice and preferences. The study conducted in the private sector of Eastern India adds value to the relative dearth of published data on the topic and the region. Since PA plays an integral part in diabetes management (American Diabetes Association, 2004), findings from the study related to PA by T2D patients are in concordance to the recent studies in India and globally (Qiu *et al*., 2012; Anjana *et al.*, 2014).

Personal or internal barriers for self-management of diabetes as stated by patients are among the common reasons as seen in previous studies. These include lack of time, laziness, weakness (Mier *et al.*, 2007; Korkiakangas *et al.*, 2009; Bryant *et al*., 2010; Miller and Marolen, 2012; Murray *et al*., 2012; Qiu *et al*., 2012). These internal barriers are individuals choices and attitude that need to be identified in order to change. Thus interventions must be planned in order for patients to first assess their areas and readiness to change, and then accordingly motivate the patient based on their acceptance of the disease and their intention to change (Fort *et al.*, 2013). Furthermore, our study highlighted important enablers such as social support by means of family and friend’s suggestions along with company for exercise, which plays an important role in self-management of diabetes. The presence of social support in the form of emotional encouragement can also help overcome laziness, tiredness, and so on, which in turn can be improved by PA. These support systems have shown to improve diabetes control, knowledge, and psychosocial functioning (Van Dam *et al*., 2005), similar to multiple sclerosis and rheumatoid arthritis patients (Aghaei *et al.*, 2016; Xu *et al*., 2017).

Higher number of males were ‘active’ than females, with no association between gender and PA uptake. A total of 27 female patients stated that ‘*they had enough work at home and felt was comparable to physical activity*’, hence did not perform any additional PA. Similar gender-based barriers have also been reported in other studies (Lawton *et al*., 2006; Mier, Medina and Ory, 2007; Bryant *et al.*, 2010).

The findings of our study showed enabling factors that motivated patients to participate in regular PA, which included ‘doctor’s advice’ as cited by 55 patients, while one patient complained lack of knowledge of exercise as a barrier. These findings further strengthen the need for counseling by treating physicians during routine visits that perhaps could also be tailored according to patients’ requirements (Nelson *et al*., 2002; Van Dam *et al*., 2005; Haskell *et al*., 2007). The harnessing of physicians to facilitate counseling recommends an imperative need for engagement of private sector providers as well as strengthening capacity of physicians across sectors.

In concordance to the principles of the Alma-Ata declaration, at primary care level for early control of a lifestyle disorder such as diabetes, adopting strategies that include inter-sectoral collaboration (of providers) and community engagement are highly recommended. Thus for achieving Health for All, use of patient-centered models for diabetes care such as the modified 3×3P rubric (Bryant *et al*., 2010) along with encouragement for weight monitoring and control by use of wearable technology (Chiauzzi, Rodarte and DasMahapatra, 2015) needs to be considered, especially for a developing country like India.
