# Type 2 Diabetes and physical activity: Barriers and enablers to diabetes control in Eastern India – ERRATUM

**DOI:** 10.1017/S1463423619000689

**Published:** 2019-09-04

**Authors:** Sanghamitra Pati, Eunice Lobo, Sandipana Pati, Shayma Desaraju, Pranab Mahapatra

**Keywords:** diabetes control, India, internal and external barriers, patient activation, personal enablers

Initials were used instead of full author names in the above mentioned article.

Cambridge University Press apologises for this error, which has since been rectified.

## References

[ref] Pati S , Lobo E , Pati S , et al. (2019) Type 2 diabetes and physical activity: barriers and enablers to diabetes control in Eastern India. Primary Health Care Research & Development 20, e44. Cambridge University Press. 10.1017/S1463423619000689 PMC6728929 31481137

